# Optogenetic inactivation of the medial septum impairs long-term object recognition memory formation

**DOI:** 10.1186/s13041-022-00938-3

**Published:** 2022-06-07

**Authors:** Maria Carolina Gonzalez, Andressa Radiske, Janine I. Rossato, Sergio Conde-Ocazionez, Lia R. M. Bevilaqua, Martín Cammarota

**Affiliations:** 1 Memory Research Laboratory, Brain Institute, Federal University of Rio Grande do Norte, Natal, RN 59078-900 Brazil; 2 Edmond & Lily Safra International Institute of Neuroscience, Macaiba, RN 59280-000 Brazil; 3 Departament of Physiology, Federal University of Rio Grande do Norte, Natal, RN 59064-741 Brazil; 4 Instituto Masira, Facultad de Ciencias Médicas y de la Salud, Universidad de Santander, Bucaramanga, Colombia

**Keywords:** Theta rhythm, Amnesia, Hippocampus, Brain oscillations, Long-term memory

## Abstract

Theta is one of the most prominent extracellular synchronous oscillations in the mammalian brain. Hippocampal theta relies on an intact medial septum (MS) and has been consistently recorded during the training phase of some learning paradigms, suggesting that it may be implicated in hippocampus-dependent long-term memory processing. Object recognition memory (ORM) allows animals to identify familiar items and is essential for remembering facts and events. In rodents, long-term ORM formation requires a functional hippocampus but the involvement of the MS in this process remains controversial. We found that training adult male Wistar rats in a long-term ORM-inducing learning task involving exposure to two different, but behaviorally equivalent novel stimuli objects increased hippocampal theta power, and that suppressing theta via optogenetic MS inactivation caused amnesia. Importantly, the amnesia was specific to the object the animals were exploring when the MS was inactivated. Taken together, our results indicate that the MS is necessary for long-term ORM formation and suggest that hippocampal theta activity is causally linked to this process.

**Supplementary Information:**

The online version contains supplementary material available at 10.1186/s13041-022-00938-3.

## Main text

**Fig. 1:**
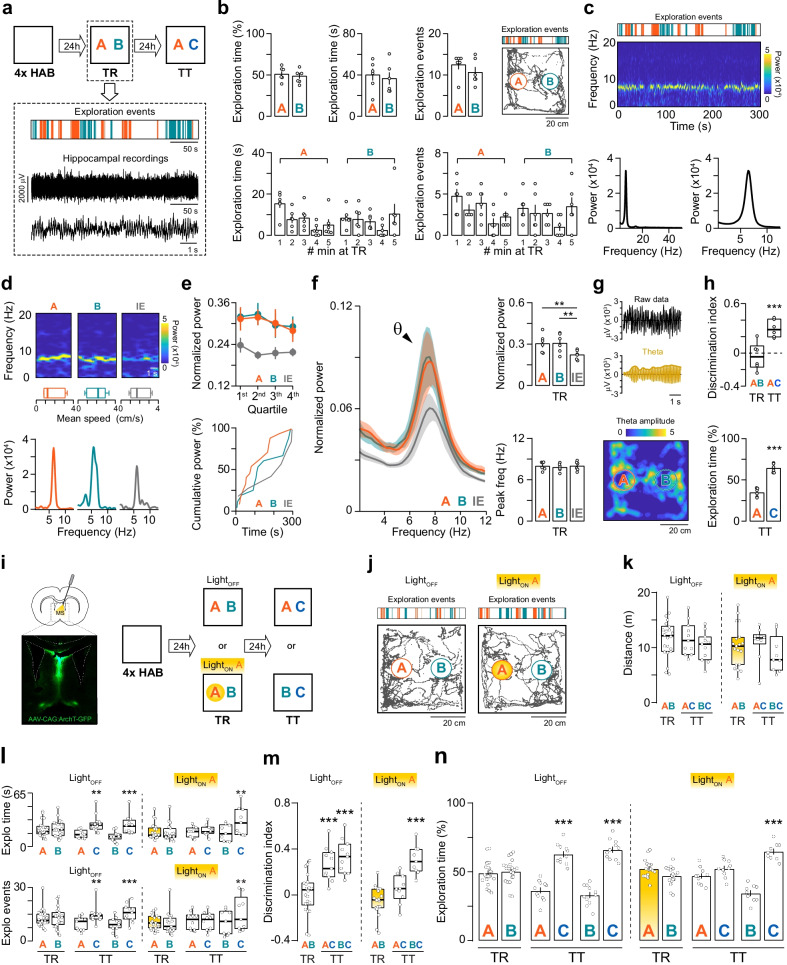
**a**–**h** Hippocampal theta activity increases during training in the novel object recognition task. Male Wistar rats implanted with electrode arrays in the CA1 region of the dorsal hippocampus (n = 6) were habituated to an open-field arena (HAB; 20-min/d/4d) and 24-h later trained in the novel object-recognition task (TR). Long-term ORM was evaluated 24-h thereafter (TT). **a** (Top) Graphic representation of the experimental design. (Bottom) Behavioral data showing exploration events distribution and hippocampal LFP recordings during TR for a representative rat. **b** (Top) Mean exploration time (% and s), mean number of exploration events, and representative trajectory during TR. (Bottom) Mean exploration time and number of exploration events per minute during TR. **c** (Top) Exploration events and theta activity during TR for a representative rat. (Bottom) Representative power spectral density plots during TR. **d** (Top) Spectrograms highlighting theta activity during exploration and inter-exploration (IE) events. (Middle) Mean locomotion speed during exploration events and IE. (Bottom) Representative power spectral density plots for exploration events and IE. **e** (Top) Mean theta power for exploration events and IE computed for interquartile intervals. (Bottom) Representative plot showing cumulative theta power during TR. **f** (Left) Mean power spectral densities for exploration and IE during TR. (Top right) Mean theta power and (Bottom right) mean theta peak frequency during TR. **g** (Top) Raw data, filtered theta and theta envelope for a representative exploration event. (Bottom) Spatial distribution of theta during TR for a representative rat. **h** (Top) Discrimination index during TR and TT. (Bottom) Mean exploration time (%) during TT. **i**–**n** Optogenetic inactivation of the medial septum causes amnesia. Two groups of rats expressing Arch-T in MS (Light_OFF_, n = 22 and Light_ON_ A, n = 19) were trained in the novel object recognition task exactly as in A, except that Light_ON_ A animals received yellow light (565-nm) stimulation during object A exploration events whereas Light_OFF_ animals were not stimulated. Long-term ORM was evaluated 24-h later. **i** Graphic representation of the experimental design. **j** Trajectory during TR for representative Light_OFF_ and Light_ON_ A rats. **k** Mean distance travelled during TR and TT. **l** Mean exploration time (s) and number of exploration events during TR and TT. **m** Discrimination index during TR and TT. **n** Mean exploration time (%) during TR and TT. Individual values in Additional file 1: Tables S1, S2

Neural oscillations are repetitive rhythmic patterns of electrical activity that occur spontaneously or in response to stimuli. Theta is a slow (5–10 Hz) neural oscillation predominantly found in the hippocampus, particularly in the CA1 region, where it is more regular and shows maximum amplitude [1]. Hippocampal theta is sensitive to medial septum (MS) lesions [2] and, although its behavioral correlates have not yet been fully elucidated, extensive evidence indicates that it supports learning [3–5]. Indeed, theta facilitates hippocampal long-term potentiation (LTP) [6], the main cellular model of hippocampus-dependent long-term memory. Object recognition memory (ORM) allows animals to determine the familiarity of items and is vital for remembering events and planning actions. In rodents, training in an ORM-based learning paradigm activates several plasticity-related signaling pathways and induces LTP in dorsal CA1, indicating that the hippocampus is essential for long-term ORM formation [7–9]. Conversely, the participation of the MS in this process remains controversial. For example, septal lesions that impair spatial and working memory do not affect long-term ORM [10, 11] but MS stimulation attenuates the long-term ORM deficit observed in epileptic mice by increasing hippocampal theta activity [12]. Therefore, we set out to analyze whether MS-regulated hippocampal theta is indeed associated with long-term ORM retention. Firstly, we determined whether long-term ORM formation affects hippocampal theta. To do that, we implanted electrode arrays in the dorsal CA1 region of adult male Wistar rats (3-months-old, 300–350 g) and trained them in the novel object-recognition paradigm, a long-term ORM-inducing task based on the rodents’ natural predilection for novelty that involves exposure to two different but behaviorally equivalent novel objects A and B in a familiar open field arena for 5-min (Fig. 1a) [13]. A digital video camera fixed above the arena was utilized for tracking, recording, and analyzing the animals’ position and behavior with the ObjectScan system software (for details see Additional file 1). Exploration events were defined as the ≥ 0.5-s-long epochs during which the animals sniffed and/or touched the stimuli objects with their muzzle and/or forepaws. All other epochs ≥ 0.5 s in duration were regarded as inter-exploration events and, of these, we further considered only those during which the mean locomotion speed was ≤ the mean locomotion speed of all exploration events. Events lasting < 0.5-s were excluded from analysis. Local field potentials (LFP) were recorded continuously during the training session. Signals were amplified, digitized, filtered at cutoff frequencies of 0.3 and 250 Hz, and sampled at 1 kHz. Data from time windows corresponding to exploration and inter-exploration events were extracted and analyzed offline using built-in or custom-written routines (see Additional file 1 for details). As expected, the exploration time and the number of exploration events during training did not differ between objects A and B (Fig. 1b; t (5) = 0.79, P = 0.46 for exploration time; t (5) = 1.21, P = 0.28 for exploration events in paired t test). Exploratory activity was observed all through the training session (Fig. 1b). Theta activity was also evident throughout this session (Fig. 1c), but theta power, which predicts learning [14], was particularly high during object exploration (Fig. 1d, e). Indeed, power spectra analysis showed that theta power during object exploration epochs was 36 ± 7% higher than during inter-exploration periods (Fig. 1f, g; F (2, 10) = 15.55; P = 0.0009. Obj A vs IE, P = 0.002, Obj B vs IE, P = 0.001 in Bonferroni’s multiple comparisons test after RM one-way ANOVA). Theta peak frequency did not differ between exploration and inter-exploration events (Fig. 1f; F (2, 10) = 3.29; P = 0.079 in RM one-way ANOVA). Neither the power nor the peak frequency of theta differed between object A and object B exploration epochs (Fig. 1f; Obj A vs Obj B, P > 0.99 for theta power; Obj A vs Obj B, P = 0.13 for peak frequency in Bonferroni’s multiple comparisons test after RM one-way ANOVA). One day after training, long-term ORM retention was evaluated by re-exposing animals to familiar object A and novel object C. As expected, the animals preferentially explored the novel object at test (TT; Fig. 1h; t (5) = 6.95, P = 0.0009 in one sample t test with theoretical mean = 50).

Normal MS functioning is essential for hippocampal theta activity [2]. In fact, MS inactivation has been used before as a tool to abolish hippocampal theta during learning [15]. Previously, we showed that yellow light (565 nm) stimulation of the MS of rats expressing the yellow light-sensing optical neural silencer archaerhodopsin T (ArchT; see Additional file 1 for technical details) [16] rapidly and reversibly cancels theta in dorsal CA1 [17]. Therefore, to analyze the involvement of the MS in long-term ORM formation and to further assess whether hippocampal theta is indeed linked to this process, rats expressing ArchT in the MS were trained in the novel object-recognition paradigm using A and B as stimuli objects, and yellow light was delivered to the MS just during object A exploration (Fig. 1i). This procedure did not affect locomotor activity (Fig. 1j, k; t (39) = 1.29, P = 0.20 for Ligth_OFF_ vs Light_ON_ A in unpaired t test), object exploration time (Fig. 1l; t (39) = 1.33, P = 0.18 for Ligth_OFF_ vs Light_ON_ A in unpaired t test), or the number of exploration events (Fig. 1l; t (39) = 1.93, P = 0.06 for Ligth_OFF_ vs Light_ON_ A in unpaired t test). Long-term ORM was evaluated during a retention test session in the presence of familiar object A or familiar object B alongside novel object C carried out 24-h post-training. We found that unstimulated ArchT-expressing animals discriminated objects A and B from novel object C (Fig. 1m, n; t (10) = 5.96, P < 0.0001 for test AC, t (10) = 7.48, P < 0.0001 for test BC in one sample t test with theoretical mean = 50); however, rats that had been delivered yellow light on the MS during object A exploration at training discriminated object B but not object A from novel object C at test (Fig. 1m, n; t (9) = 1.38, P = 0.19 for test AC, t (8) = 7.30, P < 0.0001 for test BC in one sample t test with theoretical mean = 50).

Hippocampal theta amplitude depends on locomotion speed [18], but it is unlikely that changes in this variable could account for the increase in theta power that we observed during training because we only compared exploration events with inter-exploration events matched for similar speed. It is also unlikely that the amnesia triggered by MS inactivation was due to impaired recall, subpar training performance, optogenetic construct overexpression, or a harmful effect of light stimulation per se, because it was specific to the object the animals were exploring when optogenetic suppression was applied, and light delivery did not affect object exploration. The MS not only projects to the hippocampus but also to the anterior cingulate cortex (ACC) [19]. Therefore, the amnesia induced by MS inactivation could potentially be caused by impairment of this interaction. However, the ACC is not involved in long-term ORM formation [20] and inhibition of MS-ACC projections does not affect this form of declarative-like memory [21]. Hence, it is implausible that disruption of ACC function could account for our results which are likely due to hippocampal theta inhibition. The notion that the hippocampus is required for ORM processing has received wide experimental support, but it is not unanimously accepted [22]. For example, pre-training intra-hippocampal muscimol administration affects ORM only when the training-test interval is longer than 10 min [23], suggesting that the hippocampus is not required for short-term ORM recall, that other brain regions take over the role of the hippocampus in short-term ORM processing when it remains disabled for a long time, or that short-term and long-term ORM involve independent mechanisms, as it has been reported for other memory types [24]. In this regard, our data indicate that the hippocampus is key for long-term ORM formation and substantiate further the idea that the two long-term object memories acquired during training in the novel object recognition task are independent [13]. Furthermore, the fact that the animals were amnesic only for the object they were exploring when the MS was inactivated strongly indicates that theta is not just a byproduct of learning-induced neural plasticity but is functionally linked to the calculations that occur in the hippocampus during long-term ORM formation.

## Supplementary Information

**Additional file 1.** Extended materials and methods and datasets.

## Data Availability

Please contact the corresponding author for data requests.

## Abbreviations

CA1: Cornu Ammonis-1
LFP: Local field potentials
LTP: Long-term potentiation
ORM: Object recognition memory
TT: Test
