# Supplementary material for: Optogenetic inactivation of the medial septum impairs long-term object recognition memory formation
Source: Mol Brain. 2022 Jun 7;15:50. doi: 10.1186/s13041-022-00938-3 (PMC9172102; doi:10.1186/s13041-022-00938-3)
Supplement: Supplementary file 1 — Additional file 1. Extended materials and methods and datasets. [file 13041_2022_938_MOESM1_ESM.pdf]

Optogenetic inactivation of the medial septum impairs long-term object recognition memory formation

Maria Carolina Gonzalez[1,2,§], Andressa Radiske[1,2,§], Janine I. Rossato[1,3], Sergio Conde-Ocazionez[4], Lia R.M. Bevilaqua[1], and Martín Cammarota[1,#]

[1]Memory Research Laboratory, Brain Institute, Federal University of Rio Grande do Norte, 59078-900 Natal/RN, Brazil; [2]Edmond & Lily Safra International Institute of Neuroscience, 59280-000 Macaiba/RN, Brazil; [3]Departament of Physiology, Federal University of Rio Grande do Norte, 59064-741 Natal/RN, Brazil; [4]Universidad de Santander, Facultad de Ciencias Médicas y de la Salud, Instituto Masira, Bucaramanga, Colombia. [§]Equally contributed. [#]Corresponding author at martin.cammarota@neuro.ufrn.br

Materials and Methods

Subjects

In this study, we utilized male Wistar rats (3-month-old; 300-350 g) that were housed in groups of 5, had free access to food and water and were maintained at 23-25 °C on a 12-h light/dark cycle (lights on at 06:00 AM) in the institutional vivarium. Experiments were performed during the light phase of the cycle. All procedures followed the USA National Institutes of Health Guidelines for Animal Care and were approved by the local institutional ethics committee (Comissão de Ética no Uso de Animais - Federal University of Rio Grande do Norte).

Stereotaxic surgery

Animals were anesthetized with a mixture of ketamine (80 mg/kg) and xylazine (10 mg/kg) and received micro-infusions (0.5 μl) of the adeno-associated viral vector AAV-CAG-ArchT-GFP (UNC Vector Core; 2 $\times 10^{11}$ particles/ml; serotype 2) at three different depths in the medial septum using an infusion pump (AP, -0.2; LL, -1.1; DV, - 5.5/-6.0/-6.5 in mm; 10° angle insertion), as in [1]. Two weeks later, animals were submitted to a second surgery to implant optical fibers (200-µm diameter) in the medial septum (AP, -0.2; LL, -1.1; DV, -5.5 in mm; 10° angle insertion). Six animals were implanted with electrode arrays (50 µm tungsten wires coated with PFA) in the CA1 region of the dorsal hippocampus (AP, −3.6; LL, +3; DV, −3.6 mm). Screws in the parietal/occipital bones and dental acrylic were used to fix the implants. One of the screws was used as the ground connection. Rats received subcutaneous meloxicam (0.2 mg/kg) and were allowed to recover for 7-10 days. Rats implanted with electrodes were housed individually.

Novel object recognition task

One hour before the beginning of the experimental sessions, animals were transported from the vivarium to the experimental anteroom. The animals were habituated to an empty open field (60 cm x 60 cm x 60 cm) for 20 min per day during 4 days. The training session (TR) was carried out one day after the last habituation session by placing the animals in the open field in the presence of two different novel stimuli objects for 5 minutes. Object recognition memory retention was evaluated 1 day later by re-exposing the animals to one of the familiar objects

together with a novel one for 5 minutes in the open field (TT). The experimental room and the anteroom were acclimatized at 23-24°C. Stimuli objects were made of metal, glass, or glazed ceramic and had no innate significance for rats [2]. The open field arena and the stimuli objects were cleaned thoroughly before each trial to eliminate olfactory cues. Object exploration was defined as sniffing or touching the stimuli objects with the muzzle and/or forepaws. Digital video cameras connected to an automatic video tracking system (ObjectScan software, CleverSys; RRID: SCR_017141) were fixed above the open fields to track, record, and analyze animals' behavior. Videos were acquired at 30 frames/s.

Electrophysiological recordings

Brain oscillations were recorded using the Cerebus Neural Signal processor system (BlackRock Microsystems). Neurophysiological signals were acquired continuously at 1kHz, filtered (0.3-250 Hz), amplified, digitized, and stored for offline analyses. Data were analyzed in Matlab (MathWorks, USA) using built-in and custom-written routines from the Signal Processing Toolbox. Data from time windows corresponding to exploration and inter-exploration events were extracted and analyzed. Exploration events were defined as the ≥ 0.5-s-long epochs during which the animals sniffed and/or touched the stimuli objects with their muzzle and/or forepaws. All other ≥ 0.5 s-long epochs were regarded as inter-exploration events and, of these, only those during which the mean locomotion velocity was between the 20th and 80th percentile of the locomotion velocity distribution of exploration events were considered. Data from events lasting less than 0.5 s were excluded from analysis. We averaged and analyzed data from six neighboring recording sites located in the dorsal hippocampus. Spectrograms were calculated using 2-s sliding windows of 0.2-s-long steps (90% overlap) and computing the short Fourier transform of each window with 0.122 Hz resolution. Each LFP trace was normalized using the z-score transformation and the linear trend was removed. The power spectrum was calculated computing the magnitude of the fast Fourier transform with 0.244 Hz resolution (2^12 frequency samples). Theta (5-10 Hz) band power was quantified as the frequency-restricted area under smoothed power spectrum curve (1.22 Hz smoothing bandwidth). *Heatmaps.* For each LFP, instantaneous theta amplitude was

expressed as a function of the animal position at each point in the open field represented as a 100 x 100 grid map. Theta amplitude was computed as the magnitude of the Hilbert transform of the bandpass filtered LFP in the theta frequency range. Theta amplitude values at the time of each video frame were extracted and associated with the animal's position given by the tracking system. Only times when the animal presented a locomotion velocity between the 20th and 80th percentiles of the distribution of locomotion velocities obtained during object exploration events were considered.

Optogenetic stimulation

Before the beginning of the optogenetic experiment, animals were habituated to moving freely with the fiber-optic cable attached to the implant. Optic fibers were coupled to a yellow-light (565 nm) emitting LED (ThorLabs). A pulse of light was applied using a DC4104 LED driver (ThorLabs) every time the animal approached one of the objects (i.e. object A) during the training session. The duration of each pulse corresponded to the time that the exploration event lasted.

Immunofluorescence

At the end of the experiments, rats were transcardially perfused with 4% PFA and the brains were removed and immersed 4% PFA. One day later, brains were immersed in 30% sucrose and 48-36 h thereafter they were frozen and sectioned (50 µm slices) using a cryostat. Free-floating sections were rinsed with PBS, incubated in PBST (0.2% Triton X-100) for 1 h and with normal goat serum (10% in PBST) for 2 h at room temperature. Then, sections were incubated with anti-GFP (1:1000 dilution, RRID:AB_2630379) overnight at 4°C, washed in PBST and incubated with Alexa Fluor 488 (1:1000 dilution, RRID:AB_143165) for 2 h at room temperature. Sections were counterstained with DAPI (1:1000 dilution, RRID:AB_2629482), mounted in polysine slides, covered with Fluoromount-G mounting medium, and stored at 4°C. Images were acquired using a Leica DM5000 fluorescence microscope and a CoolSNAP HQ2 CCD Camera in 16-bit grayscale. For visualization, pseudocolor was applied (linear look-up table covered full images, Image-Pro Plus 7.0 software).

Data analysis

Behavior was analyzed using the ObjectScan software. Statistical analyses were performed using GraphPad Prism 8 software (RRID:SCR_002798). Significance was set at $P < 0.05$. The number of animals per group was based on previous reports and is indicated in the figures (dots). Subjects were randomly assigned to experimental groups. Data were analyzed using Shapiro-Wilk test, one-sample t test with theoretical mean = 0, one-sample t test with theoretical mean = 50, paired t test, unpaired test and ANOVA. Discrimination between familiar and novel objects was measured using the percentage of exploration time (Time exploring the object * 100 / Total object exploration time) and the discrimination index (DI = (Time exploring novel object – Time exploring familiar object) / Total object exploration time).

**Exploration time (s)**				**# Exploration events**				**Theta power**			**Peak frequency**		
**Training**		**Test**		**Training**		**Test**		**A**	**B**	**IE**	**A**	**B**	**IE**
**A**	**B**	**A/B**	**C**	**A**	**B**	**A/B**	**C**						
49.9	36.6	66.5	99.2	14	10	13	18	0.34	0.38	0.25	7.73	7.70	7.70
56.1	60.5	43.6	72.1	13	15	12	12	0.41	0.37	0.23	7.60	7.50	7.50
41.6	45.9	26.3	62.3	14	14	11	*17*	0.29	0.29	0.22	7.11	6.90	7.16
43.8	26.8	41	80.3	14	7	14	21	0.32	0.34	0.26	8.60	8.00	8.50
16.4	24.9	9.7	24.2	7	9	2	6	0.23	0.21	0.18	8.50	8.50	8.80
32.3	24.8	37.8	54.9	13	9	15	20	0.23	0.25	0.19	8.50	8.31	8.30

Table 1. Dataset of Figure 1a-h

		Exploration time (s)				# Exploration events				Distance	
		Training		Test		Training		Test		Training	Test
		A	B	A/B	C	A	B	A/B	C		
Light OFF	Test AC	18.99	21.29	10.78	20.85	11	14	4	14	15735.05	12178.7
		16.01	19.72	10.58	23.12	15	16	6	13	14303.71	7895.57
		12.48	12.48	10.41	15.25	8	8	6	8	11794.08	8712.47
		12.18	11.88	13.95	19.02	8	6	6	12	5221.83	9839.58
		17.35	14.88	16.95	39.84	10	8	12	16	9204.08	15366.36
		20.69	38.30	15.38	54.26	16	25	13	29	13765.6	8705.46
		23.09	27.89	20.85	29.53	17	18	15	15	12003.34	14016.79
		25.69	19.89	12.75	26.12	15	15	7	15	12325.77	9901.47
		41.08	19.71	23.77	26.55	11	11	12	12	9277.44	11257.26
		12.11	13.01	22.61	28.86	10	9	14	15	10291.07	13039.74
		17.52	13.18	14.74	23.54	13	10	11	14	9779.61	17233.96
	Test BC	43.00	48.00	22.42	32.76	15	14	10	18	15023.44	13433.54
		21.15	30.26	16.02	25.62	13	14	8	18	19121.14	10636
		35.10	31.36	3.67	15.15	12	10	2	7	12625.87	5636.89
		15.83	26.36	6.11	17.95	10	17	4	12	12979.34	7414.13
		25.10	28.40	15.58	28.16	17	24	11	19	10262.33	10985.99
		13.41	10.04	11.31	16.45	10	3	7	10	5482.12	9970.72
		34.73	17.05	23.62	54.46	30	13	16	24	13191.49	8552.22
		23.45	26.32	8.84	18.51	18	13	13	13	9109.31	11309.22
		16.88	29.06	12.82	33.38	9	20	12	22	16272.68	11963.64
		16.98	21.32	12.44	24.85	6	14	9	16	13199.85	14308.98
		8.27	7.21	12.62	16.35	7	5	7	14	6205.17	7765.36
Light ON	Test AC	10.14	15.05	21.25	23.56	6	11	14	15	9364.51	13182.13
		15.95	8.51	24.22	33.70	9	5	17	19	6190.86	14190.62
		20.49	16.25	10.35	16.63	14	8	5	6	5926.96	3503.09
		13.74	12.61	7.71	9.28	10	8	6	3	6931.43	8381.74
		10.68	11.91	11.64	16.95	4	6	6	6	10546.91	11463.15
		35.90	29.16	27.56	26.63	13	15	14	13	17740.76	12056.92
		14.21	10.00	18.52	20.79	10	5	14	13	5758.16	11343.05
		12.21	11.04	22.92	16.12	7	6	19	11	11626.52	11834.3
		13.45	13.21	17.72	14.95	9	11	7	7	6862.02	12104.42
		16.05	15.18	18.08	19.42	13	7	10	16	10769.17	11637.76
	Test BC	11.04	12.29	11.88	15.32	6	5	4	8	16272.68	7801.97
		12.61	9.84	6.47	13.38	7	7	3	6	11920.05	8566.66
		24.36	22.56	5.90	19.13	16	14	2	5	9546.82	5076.41
		33.80	30.83	26.29	61.46	17	14	18	30	10673.29	14180.23
		7.37	6.01	5.81	13.54	5	3	4	7	4341.23	7714.17
		23.46	11.75	15.95	28.99	14	6	11	12	14857.39	10280.05
		45.74	49.05	23.00	35.53	21	29	19	20	9762.91	13776.24
		13.41	15.68	30.23	45.08	8	10	16	21	10280.05	5708.77
		20.84	23.72	28.40	46.58	11	13	16	29	14857.39	6001.66

Table 2. Dataset of Figure 1i-n

References

1. A. Radiske, M.C. Gonzalez, S. Conde-Ocazionez, J.I. Rossato, C.A. Köhler, M. Cammarota. Cross-frequency phase-amplitude coupling between hippocampal theta and gamma oscillations during recall destabilizes memory and renders it susceptible to reconsolidation disruption. *J Neurosci.* 40:6398-6408. (2020).

2. J.I. Rossato, M.C. Gonzalez, A. Radiske, G. Apolinário, S. Conde-Ocazionez, L.R. Bevilaqua, M. Cammarota. PKMζ inhibition disrupts reconsolidation and erases object recognition memory. *J Neurosci*. 39, 1828-1841 (2019).
